# Hospital variation in treatment for synchronous metastatic esophageal and gastric cancer: A nationwide population‐based study in the Netherlands

**DOI:** 10.1002/ijc.35491

**Published:** 2025-06-05

**Authors:** Julie F. M. Geerts, Pauline A. J. Vissers, Bianca Mostert, Bas P. L. Wijnhoven, Brigitte C. M. Haberkorn, Marie‐Paule G. F. Anten, Camiel Rosman, Geert‐Jan Creemers, Harm Westdorp, Maurice J. C. van der Sangen, Rob H. A. Verhoeven, Grard A. P. Nieuwenhuijzen

**Affiliations:** ^1^ Department of Surgery Catharina Hospital Eindhoven The Netherlands; ^2^ Department of Surgery Erasmus Medical Centre Rotterdam The Netherlands; ^3^ Department of Surgery Radboud University Medical Centre Nijmegen The Netherlands; ^4^ Department of Research & Development Netherlands Comprehensive Cancer Organization (IKNL) Utrecht The Netherlands; ^5^ Department of Medical Oncology Erasmus Medical Centre Rotterdam The Netherlands; ^6^ Department of Medical Oncology Maasstad Hospital Rotterdam The Netherlands; ^7^ Department of Gastroenterology Franciscus Gasthuis & Vlietland Rotterdam The Netherlands; ^8^ Department of Medical Oncology Catharina Hospital Eindhoven The Netherlands; ^9^ Department of Medical Oncology Radboud University Medical Centre Nijmegen The Netherlands; ^10^ Department of Radiotherapy Catharina Hospital Eindhoven The Netherlands; ^11^ Department of Medical Oncology Amsterdam UMC, location University of Amsterdam Amsterdam The Netherlands; ^12^ Cancer Center Amsterdam Cancer Treatment and Quality of Life Amsterdam The Netherlands

**Keywords:** esophageal cancer, gastric cancer, hospital variation, metastatic

## Abstract

Care for metastatic esophageal (EC) or gastric cancer (GC) includes a large variety of treatment modalities. Data on treatment variation across centers are unknown. This study investigated treatment variation across hospitals and its effect on overall survival (OS) in the Netherlands by conducting a nationwide retrospective cohort study with population‐based data from the Netherlands Cancer Registry. Patients diagnosed with synchronous metastatic EC/GC between 2015 and 2022 were included. Multilevel logistic regression assessed treatment patterns according to hospital of diagnosis. OS was analyzed using Cox regression analysis after categorizing hospitals into tertiles based on their adjusted odds (low/medium/high) for systemic treatment (chemotherapy, targeted therapy, and immunotherapy). Among 8406 EC and 3871 GC patients, the proportion receiving systemic treatment varied substantially: 19.8%–69.6% for EC and 15.8%–81.3% for GC across hospitals. Hospital of diagnosis was significantly associated with the adjusted probability of receiving systemic treatment (*p* < .0001). Ten out of 78 EC (12.8%) and 7 out of 73 (9.6%) GC hospitals had significantly lower systemic treatment probabilities. EC patients with OS ≥4 months diagnosed at hospitals with lower probabilities had significantly worse OS compared to high‐probability hospitals (hazard ratios [HR] 0.87 [0.79–0.95] *p* = .002). GC patients from low‐probability hospitals had significantly worse OS than from medium‐ (HR 0.86 [0.76–0.96], *p* = .011) or high‐probability hospitals (HR 0.73 [0.64–0.82], *p* < .0001). In conclusion, this study showed substantial hospital variation in treatment for metastatic EC and GC. Hospital of diagnosis was not only associated with the probability of receiving systemic treatment but also OS. This reflects the challenge of ensuring equal healthcare access.

## What's New?

A range of treatment options exist for metastatic esophageal (EC) and gastric cancer (GC). Which modalities or combination thereof are optimal for these malignancies, however, remains uncertain. Here, the authors examined differences in systemic treatment for metastatic EC and GC across hospitals in the Netherlands. Analyses reveal substantial variations in treatment for both diseases. Notably, while hospital of diagnosis was significantly associated with probability of receiving systemic treatment, no associations were found for diagnosis in specialized surgical or high‐volume centers. Hospital of diagnosis was further linked to overall survival. The findings underscore the importance of improving equitable access to care.

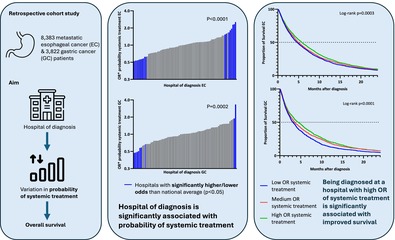

**Abbreviations**
CCI: Charlson's Comorbidity Index
EC: esophageal cancer
GC: gastric cancer
GEJ: gastroesophageal junction
HR: hazard ratio
ICC: intraclass correlation coefficient
MDTM: multidisciplinary team meetings
NCR: Netherlands' Cancer Registry
OR: odds ratio
OS: overall survival

## 1 INTRODUCTION

Almost 40% of the 2500 patients with esophageal cancer (EC) and 50% of the 1500 patients with gastric cancer (GC) present with irresectable locally advanced or synchronous metastasized disease at the time of diagnosis.^1^, ^2^, ^3^, ^4^ Even after treatment with curative intent, patients can develop metachronous metastases. Hence, the majority of patients with EC or GC will undergo palliative treatment at some point in the course of their disease.^5^

Treatment for patients with synchronous metastatic EC and GC involves a wide range of possible treatments, including systemic anti‐tumor therapy, (chemo)radiotherapy, stent placement, or supportive care. In case of a solitary lymph node or liver (oligo)metastasis, sometimes treatment with curative intent, such as (neo)adjuvant treatment followed by surgical resection and metastasectomy, is still considered.^6^, ^7^, ^8^, ^9^ A personalized treatment approach is desirable given variations in disease biology and the individual patient characteristics and preferences with optimizing quality of life (QoL) and survival as important decisive factors.^10^, ^11^ However, there are few studies that may help in selecting the optimal modality or combination of modalities for treatment in these patients.^5^, ^12^, ^13^, ^14^, ^15^, ^16^, ^17^

This lack of evidence could lead to unwanted variation in treatment and outcome between different hospitals and regions. Previous studies have demonstrated that the hospital of diagnosis is associated with the likelihood of receiving treatment with curative intent for patients with esophagogastric cancer.^18^, ^19^, ^20^, ^21^ Furthermore, a significant interhospital variation has been observed in both first and second‐line palliative systemic therapy regimens received by patients undergoing palliative treatment.^22^, ^23^

Variation in treatment of patients with palliative stage cancer in the Netherlands has been described in previous studies.^15^, ^24^ However, these studies included only patients diagnosed with EC or gastroesophageal junction cancer (GEJ) before 2017 in a limited number of hospitals in the Netherlands. The aim of the present study was to assess the present variation in treatment and survival of synchronous metastatic EC and GC in the Netherlands. We hypothesize a significant variation in systemic treatment according to hospital of diagnosis adjusted for patient and tumor characteristics. Moreover, we hypothesize that the probability of systemic treatment at a hospital is associated with survival. Knowledge of possible variation in treatment is an essential first step to ensure equal access to care.

## 2 METHODS

### 2.1 Study design and population

A retrospective, nationwide cohort study was performed. Data were obtained from the Netherlands' Cancer Registry (NCR).^25^ The NCR is a nationwide population‐based registry that includes all Dutch patients diagnosed with cancer. It is based on automatic notification of the Dutch automated pathology archive (PALGA). Additional notification from Dutch Hospital Data ensures inclusion of all not pathologically confirmed patients. Trained data managers of the NCR obtain further information on patient and tumor characteristics and treatment from medical records. Information on vital status is obtained by annual linkage to the Dutch Personal Records Database. Follow‐up was complete until February 2024. Only 14 patients (0.1%) were lost to follow‐up due to emigration.

All patients diagnosed with synchronous metastatic (cM1) esophageal or GC in the Netherlands between 2015 and 2022 were included in this study, since Dutch guidelines on first‐line palliative treatment remained largely the same during this period. Patients diagnosed abroad, neuroendocrine tumors, or neuroendocrine carcinomas were not included in the study (Figure S1). Tumor staging was registered following the Union for International Cancer Control (UICC) seventh and eighth Tumor‐Node‐Metastasis (TNM) staging manual according to the year of incidence.^26^, ^27^ Tumor location was coded according to the International Classification of Diseases for oncology (ICD‐O3).^28^ Patients with GEJ or cardia carcinoma (ICD C16.0) were categorized as EC (C15.0‐C16.0). The ICD‐O3 categories C16.1‐C16.9 were categorized as GC. Analyses were done for EC and GC separately because of varying complaints and subsequent treatment patterns associated with metastatic EC and GC.

### 2.2 Treatment modalities

Patients were categorized into mutually exclusive groups in the following order: chemoradiotherapy (CRT), systemic therapy (chemotherapy, targeted therapy, and immunotherapy), (palliative) surgical resection of the primary tumor, radiotherapy on the primary tumor, radiotherapy for metastases, surgical bypass, and stent placement. If patients did not receive any of the aforementioned treatments, they were categorized as other/no treatment. Categorization was based on the outcome of choice and invasiveness/extensiveness of treatments.

### 2.3 Hospital and region of diagnosis

The hospital of diagnosis was defined as the first institution of contact for EC or GC (*n* = 81). Since this study focused on decision‐making processes prior to the initiation of different treatments, the hospital of diagnosis was used instead of the hospital where treatment was actually administered. Hospitals were excluded from the analyses regarding the hospital of diagnosis only if fewer than 10 patients were diagnosed during the study period for EC and GC separately. Three hospitals were excluded because of this reason for EC (*n* = 23 patients), and eight hospitals for GC (*n* = 49).

Hospitals were categorized into regions based on their referral patterns. In the Netherlands, oncological regions for EC and GC are typically organized around specialized surgical centers for EC and/or GC. Physicians in such a region usually closely collaborate in regional multidisciplinary meetings and patient treatment. Thus, the diagnosis and referral of curative patients for esophagectomy or gastrectomy were used to define regions. Each hospital was linked to the esophageal or gastric surgery center to which they preferentially referred their curative EC or GC patients separately. Each hospital was associated with one oncological region per year, allowing for annual variations in groupings based on referrals. One hospital was excluded from all analyses (*n* = 50 EC and *n* = 26 GC patients) because all their surgically treated patients were referred to a hospital outside of the Netherlands. Consequently, this hospital could not be connected to a Dutch oncological region. One region was excluded from the analyses for GC because fewer than 10 GC patients were diagnosed within this region during the study period (*n* = 9).

### 2.4 Hospital volume

Hospitals were categorized into quartiles based on the volume of patients diagnosed at the hospital with EC or GC during the study period. For EC, Q1 consists of hospitals that diagnosed fewer than 66 patients (19 hospitals, *n* = 752), Q2 diagnosed 66–100 patients (20 hospitals, *n* = 1593), Q3 diagnosed 101–150 patients (20 hospital *n* = 2483), and Q4 consists of hospitals that diagnosed more than 150 patients (19 hospitals *n* = 3556). For GC: Q1 ≤ 30 (17 hospitals *n* = 384), Q2 30–46 (19 hospitals *n* = 746), Q3 47–67 (18 hospitals *n* = 1005), Q4 ≥ 68 (19 hospitals *n* = 1687).

### 2.5 Outcomes and statistical analysis

Baseline characteristics were displayed using frequencies and percentages or median and interquartile ranges (IQR) when appropriate. The proportion of different treatment modalities given to patients with metastatic EC or GC was assessed according to the hospital of diagnosis and region using descriptive statistics. The probability of receiving systemic treatment was analyzed per hospital and/or region of diagnosis. Multilevel, multivariable logistic regression models with random intercepts were created with patients nested within the hospital of diagnosis (two‐level model) and/or regions (three‐level model). The dependent variable was receiving systemic treatment or not, to analyze variation in systemic treatment among hospitals of diagnosis and regions.

The following fixed effects were included in the model to assess their effect on the probability of receiving systemic treatment: sex, age, tumor location (EC), histology (EC), differentiation grade, Lauren classification (GC), clinical T‐stage at diagnosis, clinical N‐stage at diagnosis, number of locations with metastases, being diagnosed at a specialized surgical center or not, being diagnosed at a high‐volume center or not (in quartiles), World Health Organization (WHO) performance status, and weighted Charlson's Comorbidity Index (CCI) score.^29^ Because of the aim to describe real‐world circumstances, missing data were included in the analyses as “unknown.” First, variables were added to the model univariably. When they reached univariable significance (*p* < .05), they were added to the final model. Odds ratio's (ORs) with 95% confidence intervals (95% CI) were presented based on deviation from the average odds of all hospitals, as is typical with multilevel analysis. Intraclass correlation coefficients (ICC) were calculated for the two‐level model by using the covariance parameter estimates (ICC = (estimate/[estimate + 3.29]) × 100%).^30^

Hospitals were divided into three groups based on tertiles with the highest to lowest adjusted ORs of undergoing systemic treatment to assess the association between the probability of systemic treatment and survival (EC: low OR <0.87, medium 0.87–1.13, high ≥1.13; GC: low OR <0.88, medium 0.88–1.16, high ≥1.16). Overall survival (OS) was calculated in months from the date of diagnosis to the date of death or end of follow‐up. Survival was calculated using Kaplan–Meier curves. Differences between the three groups of hospitals with low, medium, or high probability of systemic treatment were analyzed using the log‐rank test. In addition, Cox regression analysis was performed to adjust for residual confounding for which hazard ratios (HR) with 95% CI are presented. Variables added to the Cox regression model were the same that were added to the multilevel model. Proportional hazard assumptions for these variables were evaluated by generating time‐dependent covariates based on survival time. Schoenfeld residual plots were visually examined for significant interaction terms. If the covariate residuals varied over time, the variables were considered nonproportional, and the model was stratified for these variables. If the proportional hazard assumptions were violated for the variable of interest (low, medium, or high probability of systemic treatment), separate models were applied to time intervals where the assumptions were satisfied, prioritizing the longest possible period for survival analysis. *p*‐Values below .05 were considered statistically significant. Analyses were performed in SAS version 9.4 (SAS Institute, Cary, NC).

## 3 RESULTS

### 3.1 Baseline characteristics

In total, 12,277 patients were included. Of these, 8406 patients were diagnosed with EC (including 1919 [22.8%] patients with a GEJ tumor) and 3871 patients with GC (Table 1). The median age of patients with EC and GC was 69 (IQR 62–76) and 72 years (IQR 62–79), respectively. Most patients presented with one metastatic location (EC *n* = 4443, 52.9%; GC *n* = 2385, 61.6%), and 4.9% of EC patients (*n* = 414) and 11.3% of GC patients (*n* = 439) had a cT4b tumor.

**TABLE 1:** Patient and tumor characteristics.

Characteristics	All patients (*n* = 12,277, %)	Esophageal cancer (*n* = 8406, %)	Gastric cancer (*n* = 3871, %)
Sex			
Male	8792 (72.6)	6489 (77.2)	2303 (59.5)
Age (median [IQR])	70 (62–77)	69 (62–76)	72 (62–79)
Tumor location			
Esophagus	6487 (52.8)	6487 (77.2)	0
Gastroesophageal junction	1919 (15.6)	1919 (22.8)	0
Gastric (non‐cardia)	3871 (31.5)	0	3871 (100)
Tumor type			
Adenocarcinoma	10,209 (83.2)	6603 (78.6)	3606 (93.2)
Squamous cell carcinoma	1357 (11.1)	1352 (16.1)	5 (0.1)
Other	711 (5.8)	451 (5.4)	260 (6.7)
Differentiation grade			
Well/moderately differentiated (G1/G2)	3033 (24.7)	2475 (29.4)	558 (14.4)
Poorly/undifferentiated (G3/G4)	4628 (37.7)	3107 (37.0)	1521 (39.3)
Grade cannot be assessed (Gx)	4616 (37.6)	2824 (33.6)	1792 (46.3)
Lauren classification			
Intestinal	4058 (33.1)	2290 (35.6)	1068 (27.6)
Diffuse	2832 (23.1)	1052 (12.5)	1780 (46.0)
Mixed	303 (2.5)	175 (2.1)	128 (3.3)
Unknown	1710 (13.9)	815 (9.7)	895 (23.1)
Not applicable	3374 (27.5)	3374 (40.1)	0
Clinical T‐stage			
cT1/cT2	2117 (17.2)	1553 (18.5)	564 (14.6)
cT3	5686 (46.3)	4247 (50.5)	1439 (37.2)
cT4A	605 (4.9)	316 (3.8)	289 (7.5)
cT4b	853 (6.9)	414 (4.9)	439 (11.3)
cTx	3016 (24.6)	1876 (22.3)	1140 (29.4)
Clinical N‐stage			
cN0	2181 (17.8)	1023 (12.2)	1158 (29.9)
cN+	9093 (74.1)	6907 (82.2)	2186 (56.5)
cNx	1003 (8.2)	476 (5.7)	527 (13.6)
Number of locations with metastases			
1	6828 (55.6)	4443 (52.9)	2385 (61.6)
2	3314 (27.0)	2353 (28.0)	961 (24.8)
≥3	2102 (17.1)	1586 (18.9)	516 (13.3)
Unknown	33 (0.3)	24 (0.3)	9 (0.2)
WHO performance status			
0	2550 (20.8)	1881 (22.4)	669 (17.3)
1	3435 (28.0)	2488 (29.6)	947 (24.5)
2–4	2538 (20.7)	1707 (20.3)	831 (21.5)
Unknown	3754 (30.6)	2330 (27.7)	1424 (36.8)
Charlson Comorbidity Index (weighted)			
0	5790 (47.2)	3964 (47.2)	1826 (47.2)
1	3522 (28.7)	2450 (29.1)	1072 (27.7)
≥2	2522 (20.5)	1715 (20.4)	807 (20.8)
Unknown	443 (3.6)	277 (3.3)	166 (4.3)

### 3.2 Variation in treatment

**FIGURE 1:**
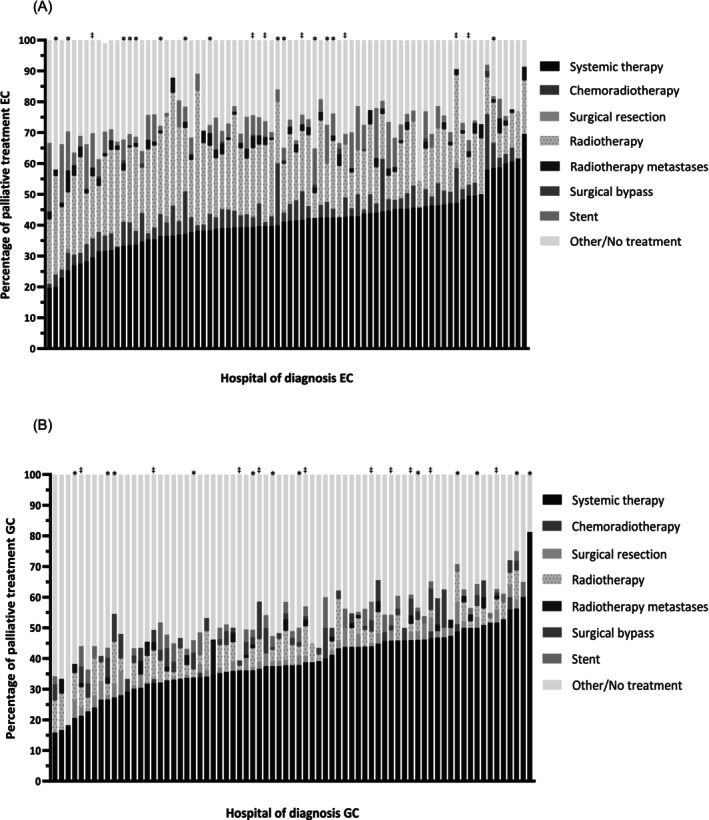
Treatment of patients with metastatic esophageal cancer (EC) (A) and gastric cancer (GC) (B) according to hospital of diagnosis. Hospitals that diagnosed <10 patients during the study period were excluded. *Specialized surgical center during entire study period. ^‡^Specialized surgical center during part of the study period.

For EC, 78 hospitals were included (*n* = 8383 patients). The proportion of patients receiving systemic therapy as their primary treatment modality varied between 19.8% and 69.6% according to hospital of diagnosis (Figure 1). CRT was applied in 0.0%–20.0% and primary resection in 0.0%–2.2% of patients. Primary treatment of radiotherapy on primary tumor and stent placement varied from 7.2% to 43.6% and 0.0% to 22.2%, respectively. Patient proportions receiving other/no treatment varied from 8.0% to 45.7%.

**FIGURE 2:**
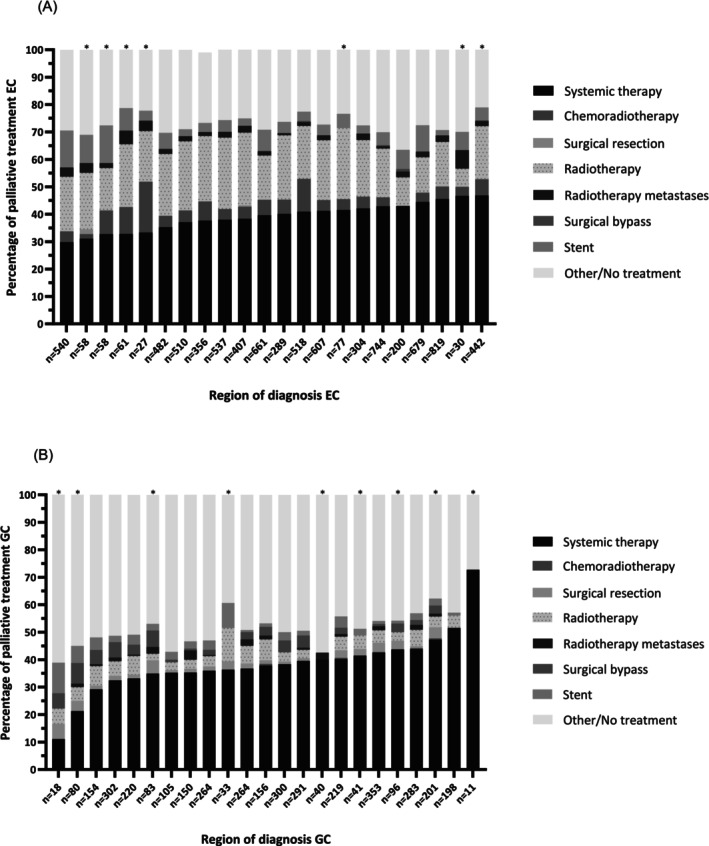
Treatment of patients with metastatic esophageal cancer (EC) (A) and gastric cancer (GC) (B) according to region of diagnosis. Regions that diagnosed <10 patients during the study period were excluded. *Region dissolved during study period due to centralization.

Hospitals were grouped in 22 oncological regions for EC. Seven of these 22 regions dissolved during the study period due to centralization. EC systemic treatment as primary treatment ranged from 29.7% to 46.8% and CRT from 0.0% to 18.5% according to oncological region (Figure 2). Radiotherapy and stent placement varied between 6.7% and 26.5% and 2.7% and 13.8%, respectively. The proportion of patients with EC receiving other/no treatment showed a variation of 15.2% between regions (20.8%–36.0%).

For GC, 74 hospitals were included (*n* = 3822) in the hospital analyses. Systemic therapy as primary treatment modality ranged from 15.8% to 81.3% between hospital of diagnosis and primary resection from 0.0% to 8.0% (Figure 1). Overall, more GC patients received other/no treatment (18.8%–81.8%) compared to EC.

All hospitals were grouped into 23 regions for GC, of which 10 dissolved during the study period due to centralization. Regional variation of more than 60.0% was observed between the lowest and highest region in the proportion of patients receiving systemic therapy as primary treatment for GC (11.1%–72.7%; Figure 2). These regions with the lowest and highest proportion of systemic therapy were also the regions that diagnosed the lowest number of patients (18 patients and 11 patients respectively). After excluding these two regions, variation in systemic therapy ranged from 21.3% to 51.5%. The proportion of primary resections ranged from 0.0% to 5.6%. Other/no treatment for patients with GC according to region of diagnosis varied more than 30.0% (27.3%–61.1%).

### 3.3 Variation in the probability of receiving systemic treatment

**FIGURE 3:**
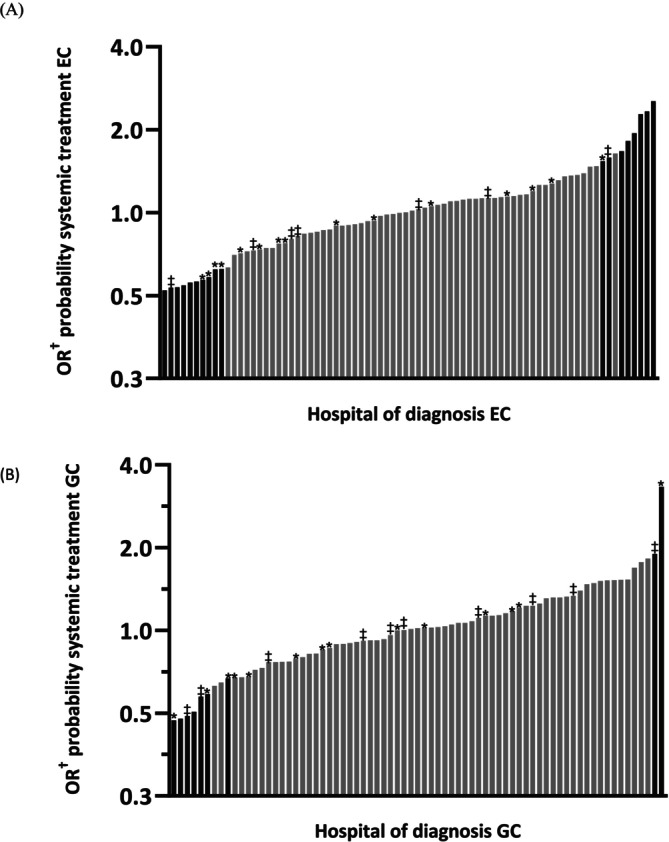
Adjusted odds ratios on the probability of receiving systemic treatment (i.e., chemotherapy, targeted therapy, and immunotherapy) according to hospital of diagnosis for esophageal cancer (EC) (A) and gastric cancer (GC) (B). Bars in black represent the hospitals with significantly lower or higher odds (*p* < .05). Hospitals that diagnosed <10 patients during the study period were excluded. * Specialized surgical center during the entire study period. ‡ Specialized surgical center during part of the study period. Adjusted for sex, age, WHO performance status, weighted Charlson Comorbidity Index score, histology (only EC), differentiation grade, Lauren classification (only GC), clinical T‐ and N‐stage, and number of locations with metastases.

Among patients with EC, 40.2% received systemic treatment as their primary treatment modality (*n* = 3368). Being diagnosed in a specialized surgical center and volume were univariably not associated with the probability of systemic treatment for EC and GC and thus not added in the multivariable models (Tables S1 and S2). The hospital of diagnosis was significantly associated with receiving systemic treatment for EC in the final model (ICC 5.1% *p* < .0001). Adjusted ORs for undergoing systemic treatment as primary treatment varied from 0.52 (95% CI 0.36–0.76) to 2.5 (95% CI 1.67–3.85) between the hospitals of diagnosis (Figure 3). Ten hospitals demonstrated a significantly lower probability of using systemic treatment when compared to the average probability of all included hospitals for EC, whereas eight hospitals showed significantly higher probabilities.

Similar to EC, 38.5% of GC patients received systemic treatment as their primary treatment modality (*n* = 1473). For GC, the hospital of diagnosis was also significantly associated with receiving systemic treatment (ICC 6.1% *p* = .0002). Adjusted ORs ranged from 0.47 (95% CI 0.25–0.90) to 3.28 (95% CI 1.73–6.21) between hospitals, with seven hospitals showing significantly lower probabilities and two significantly higher probabilities (Figure 3). Interestingly, two hospitals showed low probabilities of systemic treatment for both EC and GC, while one hospital had high probabilities for both.

In a two‐level multilevel multivariable model with region as a random intercept, the region of diagnosis was significantly associated with receiving systemic treatment (EC *p* = .02, GC *p* = .02). However, when adding both hospital and region of diagnosis as random intercepts to a three‐level model, this effect did not persist (EC *p* = .22, GC *p* = .09), while hospital of diagnosis remained significantly associated (EC *p* < .0001, GC *p* = .001). Other predictors for the lower or higher probability of receiving systemic therapy as a primary form of treatment can be found in Tables S1 and S2.

### 3.4 Survival

**FIGURE 4:**
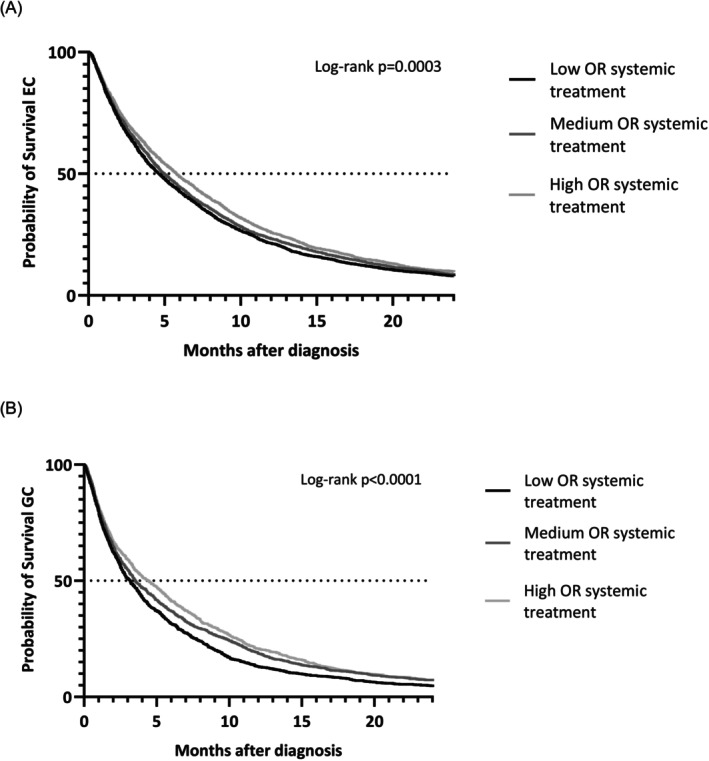
Overall survival of patients with metastatic esophageal cancer (EC) (A) or gastric cancer (GC) (B) diagnosed at hospitals with low odds, medium odds, or high odds of systemic treatment (i.e., chemotherapy, targeted therapy, and immunotherapy). Patients diagnosed at hospitals that diagnosed <10 patients during the study period were excluded. OR, odds ratio.

Median OS for all EC patients was 5.1 months (95% CI 4.8–5.3). OS was 4.6 months (95% CI 4.3–4.9) in hospitals with a low probability of undergoing systemic treatment, 5.0 months (95% CI 4.7–5.3) in hospitals with a medium probability, and 5.8 months (95% CI 5.4–6.2) in hospitals with a high probability (*p* < .0001) (Figure 4). For GC, total median OS was 3.6 months (95% CI 3.4–3.8). OS was 3.1 months (95% CI 2.8–3.4) in low‐probability hospitals, 3.9 months (95% CI 3.5–4.4) in medium‐probability hospitals, and 4.4 months (95% CI 3.8–5.0) in high‐probability hospitals (*p* < .0001) (Figure 4).

The proportional hazards assumption did not hold for the variable of interest (low/medium/high probability) for EC for the entire study period. Patients were split into two groups: those with survival <4 months and those with survival ≥4 months. The assumption held for both these groups separately for the variable of interest (Table S3). Within the first group, patients diagnosed in a hospital with medium or high probability of receiving systemic treatment did not have better OS than patients in a low‐probability hospital (medium HR 0.94, 95% CI 0.87–1.02, *p* = .13; high HR 0.94, 95% CI 0.86–1.04, *p* = .22). For patients with survival ≥4 months, being diagnosed in a high‐probability hospital was significantly associated with OS (HR 0.86, 95% CI 0.79–0.95, *p* = .002), while a medium‐probability hospital was not (HR 0.92, 95% CI 0.85–1.00, *p* = .06; Table 2). For GC, the proportional hazards assumption held for the entire study period. Multivariable Cox regression analysis confirmed that GC patients diagnosed in a hospital with a medium or high probability of receiving systemic treatment had better OS than patients in a low‐probability hospital (medium HR 0.86, 95% CI 0.76–0.96, *p* = .01; High HR 0.73, 95% CI 0.64–0.82, *p* < .0001) during the entire study period (Tables 2 and S3).

**TABLE 2:** Results of 1‐year overall survival (OS) and Cox regression analyses of OS for patients with metastatic esophageal (EC) or gastric cancer (GC) according to the adjusted odds (OR) of undergoing systemic treatment at the hospital of diagnosis.

	Probability of receiving systemic treatment, OR	Median OS, months (95% CI)	*p*‐Value	1‐year OS, %	*p*‐Value	Multivariable HR^a^ (95% CI)	*p*‐Value
EC	Low OR: <0.87 (*n* = 1559)	4.6 (4.3–4.9)		21.3		Ref^b^	
	Medium OR: 0.87–1.13 (*n* = 1890)	5.0 (4.7–5.3)		23.2		0.92 (0.85–1.01)^b^	.064
	High OR: ≥1.13 (*n* = 1295)	5.8 (5.4–6.2)	<.0001	25.7	.0003	0.87 (0.79–0.95)^b^	.002
GC	Low <0.87 (*n* = 1278)	3.1 (2.8–3.4)		13.7		Ref^c^	
	Medium 0.88–1.16 (*n* = 1351)	3.9 (3.5–4.4)		19.1		0.86 (0.76–0.96)^c^	.011
	High ≥1.16 (*n* = 1193)	4.4 (3.8–5.0)	<.0001	20.7	<.0001	0.73 (0.64–0.82)^c^	<.0001

*Note*: Hospitals that diagnosed <10 patients during the study period were excluded.

Abbreviations: CI, confidence intervals; HR, hazard ratio.

^a^ Adjusted for sex, age, WHO performance status, weighted Charlson Comorbidity Index score, tumor location (only EC) histology (only EC), differentiation grade, Lauren classification (only GC), clinical T‐ and N‐stage, and number of locations with metastases.

^b^ Cox regression model including patients with survival ≥4 months. Stratified for age, performance status, and number of metastases because of violations of proportional hazard assumptions.

^c^ Stratified for age, cT, cN, and Lauren classification due to violation of proportional hazard assumptions.

## 4 DISCUSSION

This nationwide population‐based study showed large interhospital variation in the care of patients with synchronous metastatic EC or GC in the Netherlands. Multilevel multivariable analysis confirmed that the hospital of diagnosis was significantly associated with the probability of receiving (palliative) systemic treatment. In addition, this study showed significantly higher OS for the group of patients diagnosed in hospitals with a high probability of potentially systemic treatment.

This study found different treatment patterns for metastatic EC and metastatic GC. Mainly, a higher proportion of patients with GC received other/no treatment with a range of 18.8%–81.8%, compared to 8.0%–45.7% for EC. One of the reasons for this variation could be that there are more treatment options available for EC. Radiotherapy and stent placement are viable options for EC to relieve symptoms, while these are hardly used for GC. In addition, the metastatic pattern of GC is different from EC.^2^ One of the most common metastatic locations for GC is the peritoneum. This specific type of metastatic pattern has an especially bad prognosis. This is reflected in the survival analysis, with a worse median OS for GC compared to EC. Patients with particularly bad prognosis are more likely to not receive (systemic) treatment.^31^

Several factors may contribute to practice variation in treatment for metastatic EC and GC.

Hospital‐level factors include individual physician's beliefs, values, and experience with specific diseases or treatment.^32^, ^33^, ^34^ Despite presumably more oncological expertise in specialized surgical centers or high‐volume centers, this study did not observe a higher likelihood of receiving potentially systemic treatment when patients were diagnosed in these centers. Physician assessments of patient suitability for potentially systemic therapy, considering factors like age, performance status, and comorbidities, may substantially influence treatment decisions. Patient preferences are the most important factor in decisions regarding treatment, yet the underlying process of preference development, including the role of physician communication and shared decision‐making, remains unexplored in the Netherlands.^34^, ^35^ Further research is needed to investigate these factors and the methods used to assess patient suitability and decisions for potentially systemic treatment.

Regional factors may also contribute to practice variation in treatment for metastatic EC and GC. Variation may be influenced by the establishment of formal regional networks, including regional multidisciplinary team meetings (MDTM) in which treatment plans are discussed as part of regional care pathways.^24^, ^36^, ^37^ Recent studies in the Netherlands found considerable variation in the number of patients discussed in MDTMs, particularly for those in palliative stages, with a significantly association with the hospital of diagnosis.^38^, ^39^ Unlike curative treatment, palliative care is likely provided closer to their residences, leading to decentralization and potentially more treatment variation due to geographical differences in patient populations.^22^, ^40^

Centralization of palliative care might mitigate practice variation. However, although a decrease of interhospital variation occurred in the years after centralization of curative treatment for EC, no significant change in variation was found for GC.^18^, ^21^ Additionally, this study showed that the region of diagnosis was not significantly associated with a higher probability of systemic treatment after accounting for the hospital of diagnosis, indicating that variation between individual hospitals within regions might be more substantial than between different regions or networks. This suggests that factors at the hospital level are a more important aspect in determining treatment variation than regional factors.

This study showed that patients diagnosed at hospitals with a low probability of receiving systemic treatment had a significantly poorer OS compared to those diagnosed at hospitals with medium (for GC) or high probabilities. This aligns with several meta‐analyses demonstrating survival benefits of palliative chemotherapy in EC and GC.^41^, ^42^ For EC, the proportional hazard assumption did not hold for the variable of interest (low/medium/high probability). This was unsurprising when considering that every group consists of all patients diagnosed in each hospital, and not just the patients that received systemic treatment. Since approximately 40% of patients died within 3 months of diagnosis and any effects from systemic treatment have not taken effect yet, it is unsurprising that within the first months, no survival difference is seen, but is seen in the later months. This was confirmed by the Cox regression analysis for EC. There is no difference in OS for EC for patients with survival <4 months in low, medium, or high‐probability hospitals. However, patients with survival ≥4 months do show significantly improved OS when diagnosed in a high‐probability hospital compared to a low‐probability hospital. In this study, median OS improved by 1.2 and 1.3 months when comparing low‐probability with high‐probability hospitals for EC and GC, respectively. Although a 1‐month survival improvement might seem modest when comparing treatment to placebo in a trial, such improvement is relevant in population‐based data in which patients receive varying levels of treatment (unlike trials in which patients either receive treatment or do not). This survival improvement significantly associated with the hospital of diagnosis is particularly noteworthy.

It is important to note that survival may not be the sole determining factor when considering treatment options for patients with metastatic EC and GC. QoL is usually equally, if not more, important in these decisions.^42^, ^43^ While QoL may be positively impacted by alleviation of tumor‐associated symptoms in case of treatment response, the associated side effects may have a negative impact on QoL. Unfortunately, this study lacked data on the QoL of the included patients. A meta‐analysis of QoL during palliative systemic therapy for EC or GC described stable global health status during treatment.^44^ This was confirmed by a recent study among Dutch patients, which found maintained or even improved QoL outcomes during first‐line systemic treatment for patients with advanced EC or GC. These findings argue in favor of a more liberal use of systemic treatment.^45^ With improved survival rates and possibly QoL, it is important to ensure that patients have equal opportunities for systemic treatment and, consequently, improved survival rates, regardless of their hospital of diagnosis.

Major strengths of this study are its large cohort and its use of real‐world data from a nationwide registry, which ensures a representative cohort of patients from a Western population. A limitation of this study is that, due to its retrospective design, missing data could introduce bias or limit the completeness of the analysis. However, the study findings indicated that unknown data were significantly associated with a higher probability of receiving no treatment. This suggests that missing data on these variables were not random but rather a result of a deliberate decision to refrain from further diagnostics due to the patients being deemed ineligible for further systemic treatment. The effect of variables with lots of “unknowns” on probability of systemic treatment or survival might in reality be larger than what was found in this study. Secondly, some variables such as reasons for refraining from systemic treatment were not registered in the NCR. Further research should focus on clarifying these reasons and the decision‐making processes. Thirdly, the probability of systemic treatment could act as a collider, which should be considered when interpreting the data. However, since the probability of systemic treatment was analyzed according to the hospital of diagnosis, we expect this effect to be small, since it is unlikely that one hospital receives patients with more favorable characteristics than another hospital in the Netherlands. Lastly, some patients with oligometastatic disease that are classified as cM1 in the NCR would still be treated with curative intent in the Netherlands, such as patients with a solitary supraclavicular lymph node or solitary liver metastasis.^6^ These patients usually have a more favorable prognosis which might have an effect on OS for some treatment groups in this study.^7^, ^8^ However, due to our large cohort and the knowledge that this concerns a small group of patients, we expect this effect to be small. Multilevel models were used to analyze the primary outcomes, because of the hierarchical structure of the data. Nesting individuals in groups allows for consideration of the context of individuals, such as the hospital at which they were diagnosed. Regular regression ignores the potential influence of this context on the individual and ignores the dependence of the individual on their context.^46^, ^47^, ^48^ However, a disadvantage of structuring data in this way is the need for a large sample size (of groups as well as individuals within these groups), which might affect outcomes or lead to more imprecise CIs.^46^, ^49^

In conclusion, this nationwide population‐based study revealed significant variation in the treatment of patients with metastatic EC or GC. The probability of receiving systemic treatment was significantly associated not only with patient‐ and tumor‐related factors, but also with the hospital of diagnosis. A higher probability correlated with a modest but statistically significant improvement in OS depending on the hospital of diagnosis. Selecting the optimal treatment plan for patients with metastatic EC or GC remains a complex challenge. Acquiring more evidence to improve guidance for patient selection and increased utilization of regional tumor networks may help reduce interhospital variation and ensure that all patients have equal access to care regardless of their hospital of diagnosis.

## AUTHOR CONTRIBUTIONS

**Julie F. M. Geerts:** Conceptualization; methodology; writing – original draft; writing – review and editing; formal analysis. **Pauline A. J. Vissers:** Conceptualization; methodology; formal analysis; writing – review and editing. **Bianca Mostert:** Writing – review and editing. **Bas P. L. Wijnhoven:** Writing – review and editing. **Brigitte C. M. Haberkorn:** Writing – review and editing. **Marie‐Paule G. F. Anten:** Writing – review and editing. **Camiel Rosman:** Writing – review and editing. **Geert‐Jan Creemers:** Writing – review and editing. **Harm Westdorp:** Writing – review and editing. **Maurice J. C. van der Sangen:** Writing – review and editing. **Rob H. A. Verhoeven:** Writing – review and editing; methodology; conceptualization; formal analysis. **Grard A. P. Nieuwenhuijzen:** Conceptualization; methodology; supervision; writing – review and editing.

## CONFLICT OF INTEREST STATEMENT

Grard A. P. Nieuwenhuijzen: consultant or advisory role at Medtronic. Camiel Rosman: consultant DEKRA medical BV, research funding from Johnson & Johnson, Medtronic, and ZonMw. Rob H. A. Verhoeven: Research grant for Bristol Myers Squibb and Amgen and consultant for Daiichi‐Sankyo, all paid to institution. Bas P. L. Wijnhoven: research funding from BMS; consulting/advisory for BMS, Medtronic. Bianca Mostert: research funding from Sanofi, Pfizer, and BMS; consulting/advisory for Lilly, Servier, BMS, Amgen, and AstraZeneca. All remaining authors have declared no conflicts of interest.

## ETHICS STATEMENT

According to the Central Committee on Research involving Human Subjects (CCMO), this retrospective observational study does not require ethics approval from an ethics committee in the Netherlands. The Privacy Review Board of the Netherlands Cancer Registry approved the use of anonymous data for this study.

## Supporting information


**Data S1.** Supporting Information.

## Data Availability

The data underlying this article were provided by the Netherlands Comprehensive Cancer Organization (IKNL). Data can be shared according to regular procedures of the Netherlands Cancer Registry (NCR) upon reasonable request.
