# Supplementary material for: Hospital variation in treatment for synchronous metastatic esophageal and gastric cancer: A nationwide population‐based study in the Netherlands
Source: Int J Cancer. 2025 Jun 5;157(7):1446–57. doi: 10.1002/ijc.35491 (PMC12334901; doi:10.1002/ijc.35491)

Supplementary material

Hospital variation in treatment for synchronous metastatic esophageal and gastric cancer: a nationwide population-based study in the Netherlands

Julie F.M. Geerts, MD[1,3], Pauline A.J. Vissers, PhD[2,3], Bianca Mostert[4], MD, PhD, Bas P.L. Wijnhoven, MD, PhD[5], Brigitte C.M. Haberkorn, MD[6], Marie-Paule G.F. Anten, MD[7], Camiel Rosman, MD, PhD[3], Geert-Jan Creemers MD[8], PhD, Harm Westdorp[9], MD, PhD, Maurice J.C. van der Sangen MD[10], PhD, Rob H.A. Verhoeven, PhD[2,11,12], Grard A.P. Nieuwenhuijzen, MD PhD[1]

Table of contents

Supplementary table 1..2
Supplementary table 2..3
Supplementary table 3..4
Supplementary figure 1 5

Supplementary table 1

Results of univariable and multivariable multilevel logistic regression analyses to identify predictors of systemic treatment in metastatic esophageal cancer (EC) in the Netherlands. Patients were nested within their hospital of diagnosis. Hospitals that diagnosed <10 patients during the study period were excluded.

	**Univariable EC (OR [95% CI]);**	***p-value***	**Multivariable EC (OR [95% CI]);**	***p-value***
**Diagnosed in expert center**				
Yes	1 [reference]			
No	1.22 (0.99-1.49)	0.056		
**Diagnosed in high/low volume center**				
Q1	1.26 (0.99-1.60)	0.054		
Q2	0.98 (0.80-1.20)	0.844		
Q3	0.99 (0.82-1.21)	0.953		
Q4	1 [reference]			
**Sex**				
Male	1 [reference]		1 [reference]	
Female	0.67 (0.60-0.75)	<0.0001	0.81 (0.71-0.92)	0.002
**Age**	0.93 (0.92-0.93)	<0.0001	0.94 (0.93-0.94)	<0.0001
**Tumor location**				
Esophagus	1 [reference]		1 [reference]	
Gastroesophageal junction	1.54 (1.39-1.71)	<0.0001	1.47 (1.30-1.67)	<0.0001
**Differentiation grade**				
G1-G2	1 [reference]		1 [reference]	
G3-G4	0. 88 (0.79-0.98)	0.021	0.82 (0.72-0.93)	0.002
Unknown/missing	0.67 (0.60-0.75)	<0.0001	0.78 (0.68-0.90)	0.0004
**Histology**				
Adenocarcinoma	1 [reference]		1 [reference]	
Squamous cell	0. 34 (0.29-0.39)	<0.0001	0.39 (0.34-0.46)	<0.0001
Other/unknown	0.19 (0.14-0.25)	<0.0001	0.36 (0.27-0.50)	<0.0001
**WHO performance status**				
0	1.57 (1.38-1.78)	<0.0001	1.28 (1.12-1.47)	0.0005
1	1 [reference]		1 [reference]	
2-4	0.20 (0.16-0.24)	<0.0001	0.23 (0.19-0.27)	<0.0001
Unknown	0.27 (0.24-0.31)	<0.0001	0.29 (0.25-0.33)	<0.0001
**Charlson Comorbidity Index (Weighted)**				
0	1 [reference]		1 [reference]	
1	0.69 (0.62-0.76)	<0.0001	0.94 (0.83-1.06)	0.322
≥2	0.40 (0.35-0.45)	<0.0001	0.67 (0.58-0.79)	<0.0001
Unknown	0.72 (0.56-0.94)	0.016	1.15 (0.84-1.56)	0.379
**cT-stage**				
cT1/cT2	1 [reference]		1 [reference]	
cT3	1.09 (0.97-1.23)	0.168	1.02 (0.89-1.18)	0.769
cT4A	1.05 (0.82-1.35)	0.705	1.01 (0.75-1.35)	0.948
cT4B	0.50 (0.39-0.64)	<0.0001	0.59 (0.44-0.79)	0.0003
cTx	0.74 (0.65-0.86)	<0.0001	0.90 (0.77-1.07)	0.232
**cN-stage**				
cN0	1 [reference]		1 [reference]	
cN+	1.29 (1.13-1.49)	0.0004	1.05 (0.89-1.23)	0.550
cNx	0.57 (0.44-0.73)	<0.0001	0.60 (0.45-0.81)	0.0007
**# of locations with metastases**				
1	1 [reference]		1 [reference]	
2	1.19 (1.08-1.32)	0.0009	1.27 (1.13-1.44)	0.0001
≥3	1.04 (0.92-1.17)	0.555	1.08 (0.94-1.24)	0.288
Unknown	0.67 (0.27-1.63)	0.373	0.59 (0.22-1.60)	0.297

Supplementary table 2

Results of univariable and multivariable multilevel logistic regression analyses to identify predictors systemic treatment in metastatic gastric cancer (GC) in the Netherlands. Patients were nested within their hospital of diagnosis. Hospitals that diagnosed <10 patients during the study period were excluded.

	Univariable GC (OR [95% CI]);	*p-value*	Multivariable GC (OR [95% CI]);	*p-value*
**Diagnosed in expert center**				
Yes	1 [reference]			
No	0.95 (0.76-1.19)	0.644		
**Diagnosed in high/low volume center**				
Q1	1.02 (0.75-1.39)	0.885		
Q2	1.18 (0.90-1.54)	0.219		
Q3	1.13 (0.87-1.45)	0.363		
Q4	1 [reference]			
**Sex**				
Male	1 [reference]			
Female	0.94 (0.82-1.08)	0.395		
**Age**	0.93 (0.92-0.93)	<0.0001	0.93 (0.92-0.93)	<0.0001
**Differentiation grade**				
G1-G2	1 [reference]		1 [reference]	
G3-G4	0.97 (0.79-1.19)	0.785	0.79 (0.61-1.03)	0.078
Unknown/missing	0.80 (0.65-0.97)	0.025	0.84 (0.64-1.08)	0.173
**Lauren classification**				
Intestinal	1 [reference]		1 [reference]	
Diffuse	1.12 (0.96-1.31)	0.155	0.78 (0.63-0.96)	0.020
Mixed	1.02 (0.70-1.49)	0.931	0.75 (0.47-1.19)	0.223
Indeterminate/unknown	0.50 (0.41-0.60)	<0.0001	0.58 (0.45-0.73)	<0.0001
**WHO performance status**				
0	1.54 (1.24-1.91)	<0.0001	1.35 (1.07-1.70)	0.012
1	1 [reference]		1 [reference]	
2-4	0.22 (0.18-0.27)	<0.0001	0.24 (0.19-0.30)	<0.0001
Unknown	0.19 (0.16-0.23)	<0.0001	0.21 (0.17-0.26)	<0.0001
**Charlson Comorbidity Index (Weighted)**				
0	1 [reference]		1 [reference]	
1	0.65 (0.55-0.76)	<0.0001	0.96 (0.79-1.16)	0.652
≥2	0.33 (0.27-0.40)	<0.0001	0.68 (0.54-0.85)	0.0009
Unknown	0.82 (0.58-1.15)	0.252	1.07 (0.70-1.63)	0.754
**cT-stage**				
cT1/cT2	1 [reference]		1 [reference]	
cT3	1.22 (0.99-1.50)	0.057	1.07 (0.84-1.38)	0.571
cT4A	1.30 (0.97-1.75)	0.074	1.03 (0.73-1.47)	0.859
cT4B	0.84 (0.65-1.10)	0.201	0.75 (0.55-1.03)	0.073
cTx	0.74 (0.60-0.92)	0.007	0.74 (0.57-0.96)	0.024
**cN-stage**				
cN0	1 [reference]		1 [reference]	
cN+	1.09 (0.94-1.27)	0.258	1.05 (0.88-1.26)	0.581
cNx	0.49 (0.39-0.63)	<0.0001	0.61 (0.45-0.81)	0.0008
**# of locations with metastases**				
1	1 [reference]			
2	1.02 (0.87-1.20)	0.789		
≥3	0.84 (0.68-1.03)	0.085		
Unknown	0.77 (0.19-3.16)	0.716		

Supplementary table 3

Results of cox regression analyses in metastatic esophageal cancer (EC) or gastric cancer (GC) in the Netherlands.

Hospitals that diagnosed <10 patients during the study period were excluded. Adjusted for sex, age, WHO performance status, weighted Charlson Comorbidity Index score, location tumor (only EC), histology (only EC), differentiation grade, Lauren classification (only GC), clinical T- and N-stage, and number of locations with metastases.

	**EC survival 0-4 months[a] (HR [95% CI]); n=3627**	***p-value***	**EC survival ≥ 4 months[b] (HR [95% CI]); n=4744**	***p-value***	**GC[c] (HR [95% CI]); n=3822**	***p-value***
**Sex**						
Male	1 [reference]		1 [reference]		1 [reference]	
Female	0.96 (0.88-1.04)	0.316	1.02 (0.93-1.11)	0.739	0.91 (0.82-1.01)	0.069
**Age**	1.01 (1.01-1.02)	<0.0001				
**Tumor location**						
Esophagus	1 [reference]		1 [reference]		NA	
Gastroesophageal junction	1.05 (0.97-1.15)	0.237	1.00 (0.92-1.09)	0.972	NA	
**Differentiation grade**						
G1-G2	1 [reference]		1 [reference]		1 [reference]	
G3-G4	1.01 (0.92-1.11)	0.867	1.20 (1.10-1.30)	<0.0001	1.31 (1.10-1.56)	0.002
Unknown/missing	0.99 (0.90-1.09)	0.775	1.05 (0.96-1.14)	0.308	1.45 (1.23-1.72)	<0.0001
**Histology**						
Adenocarcinoma					NA	
Squamous cell			0.97 (0.88-1.07)	0.553	NA	
Other/unknown			1.33 (1.04-1.69)	0.024	NA	
**WHO**						
0					0.72 (0.62-0.85)	<0.0001
1					1 [reference]	
2-4					1.80 (1.55-2.09)	<0.0001
Unknown					1.93 (1.68-2.22)	<0.0001
**Charlson Comorbidity Index (Weighted)**						
0	1 [reference]		1 [reference]		1 [reference]	
1	0.98 (0.90-1.07)	0.653	1.09 (1.00-1.18)	0.045	1.03 (0.91-1.17)	0.657
≥2	1.95 (0.86-1.04)	0.240	1.03 (0.93-1.14)	0.553	1.06 (0.92-1.21)	0.350
Unknown	0.87 (0.72-1.07)	0.183	0.98 (0.79-1.20)	0.823	0.73 (0.55-0.97)	0.037
**cT-stage**						
cT1/cT2			1 [reference]			
cT3			0.91 (0.84-1.00)	0.050		
cT4A			1.00 (0.82-1.23)	0.974		
cT4B			1.07 (0.88-1.31)	0.457		
cTx			1.14 (1.01-1.27)	0.032		
**cN-stage**						
cN0	1 [reference]					
cN+	0.92 (0.83-1.03)	0.158	1.14 (1.02-1.27)	0.016		
cNx	1.21 (1.03-1.42)	0.018	1.13 (0.91-1.40)	0.264		
**# of locations with metastases**						
1					1 [reference]	
2					1.28 (1.14-1.45)	<0.0001
≥3					1.90 (1.62-2.24)	<0.0001
Unknown					0.69 (0.22-2.12)	0.515

[a]Including patients with survival < 4 months. Stratified by histology, performance status, cT and number of locations with metastases.

[b]Cox regression model including patients with survival ≥ 4 months. Stratified by age, performance status and number of metastases because of violations of proportional hazard assumptions.

[c]Stratified by age, cT, cN, and Lauren classification due to violation of proportional hazard assumptions.

Supplementary figure 1

Flow chart of patient inclusion.

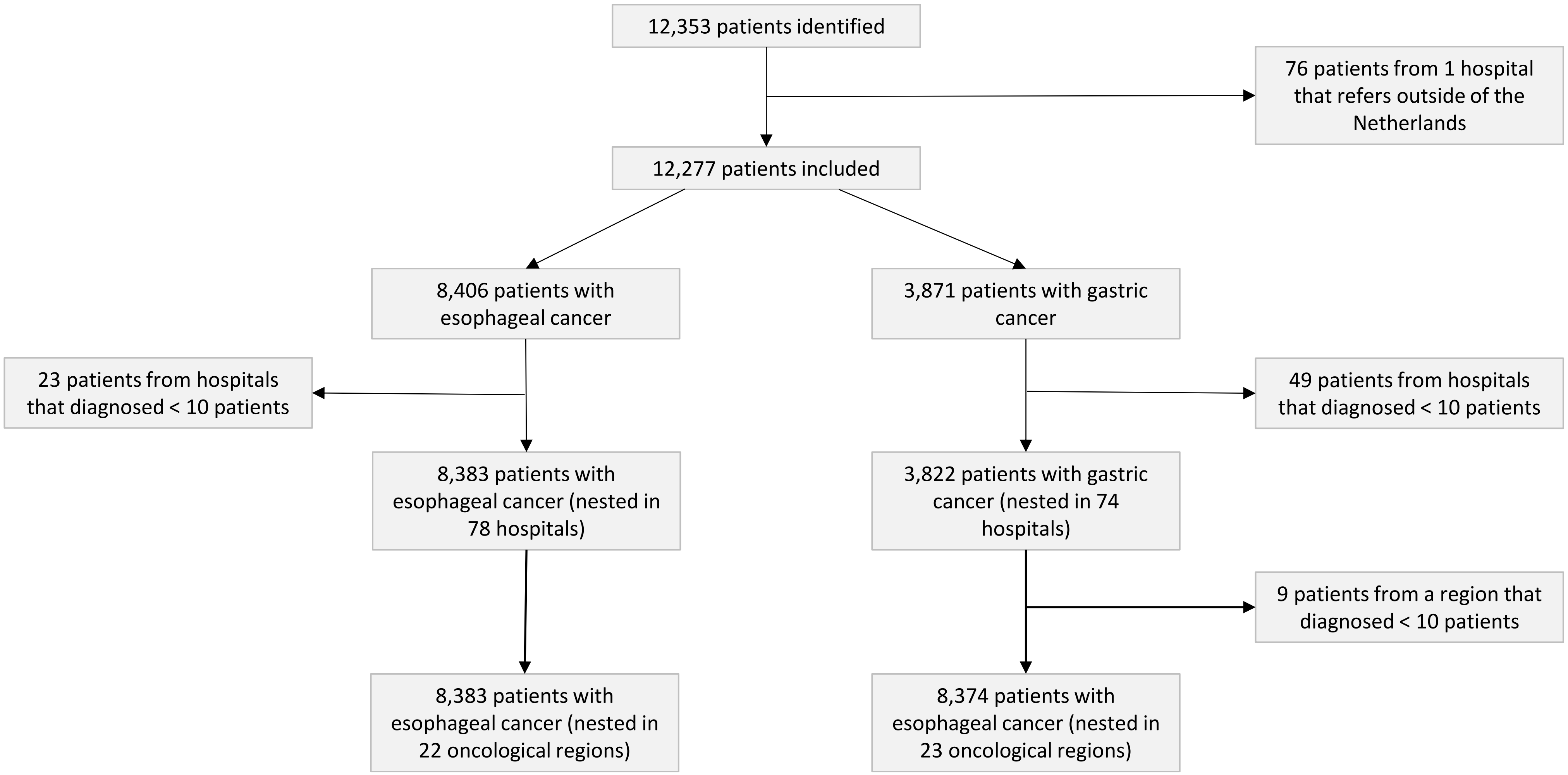

Supplement: Supplementary file 1 — Data S1. Supporting Information. [file IJC-157-1446-s001.pdf]
